# Blood alcohol concentration in the clinical laboratory: a narrative review of the preanalytical phase in diagnostic and forensic testing

**DOI:** 10.11613/BM.2024.010501

**Published:** 2023-12-15

**Authors:** Cristiano Ialongo

**Affiliations:** Department of Experimental Medicine, Policlinico Umberto I, ‘Sapienza’ University, Rome, Italy

**Keywords:** preanalytical phase, blood alcohol concentration, specimen handling, humans, substance abuse detection

## Abstract

The analysis of blood alcohol concentration (BAC), a pivotal toxicological test, concerns acute alcohol intoxication (AAI) and driving under the influence (DUI). As such, BAC presents an organizational challenge for clinical laboratories, with unique complexities due to the need for forensic defensibility as part of the diagnostic process. Unfortunately, a significant number of scientific investigations dealing with the subject present discrepancies that make it difficult to identify optimal practices in sample collection, transportation, handling, and preparation. This review provides a systematic analysis of the preanalytical phase of BAC that aims to identify and explain the chemical, physiological, and pharmacological mechanisms underlying controllable operational factors. Nevertheless, it seeks evidence for the necessity to separate preanalytical processes for diagnostic and forensic BAC testing. In this regard, the main finding of this review is that no literature evidence supports the necessity to differentiate preanalytical procedures for AAI and DUI, except for the traceability throughout the chain of custody. In fact, adhering to correct preanalytical procedures provided by official bodies such as European federation of clinical chemistry and laboratory medicine for routine phlebotomy ensures both diagnostic accuracy and forensic defensibility of BAC. This is shown to depend on the capability of modern pre-evacuated sterile collection tubes to control major factors influencing BAC, namely non-enzymatic oxidation and microbial contamination. While certain restrictions become obsolete with such devices, as the use of sodium fluoride (NaF) for specific preservation of forensic BAC, this review reinforces the recommendation to use non-alcoholic disinfectants as a means to achieve “error-proof” procedures in challenging operational environments like the emergency department.

## Introduction

Ethyl alcohol (C_2_H_5_OH, CAS 64-17-5) or ethanol (EtOH) or just “alcohol”, is a liquid organic compound at room temperature that forms the basis of various aqueous beverages (spirits, wine, or beer) consumed by half of the world’s population over the age of 15, the majority of whom reside in the Americas, Europe, and the Western Pacific (*1*). For individuals up to the age of 49, the consumption of EtOH is the leading risk factor for premature death and disability, with mortality rates even exceeding those of diabetes, tuberculosis, and human immunodeficiency virus (HIV)/acquired immunodeficiency syndrome (AIDS) (*2*).

Blood alcohol concentration (BAC) analysis is a toxicological test associated with two important conditions: acute alcohol intoxication (AAI) and driving under the influence (DUI). Acute alcohol intoxication is a clinical emergency that affects 7.5% of drinkers and causes 2200 deaths *per* year in countries such as the United States (*1*, *3*). The diagnostic determination of BAC is routinely performed by means of enzymatic reaction quantified *via* automated spectrophotometry (*i.e*., clinical chemistry auto-analysers), using serum or plasma as the elective matrix being thus referred to as serum alcohol concentration (SAC) or plasma alcohol concentration (PAC). Driving under the influence, on the other hand, is a condition that, although not necessarily associated with fatal intoxication, results in cognitive impairment responsible for 25% of fatal road accidents, causing approximately 10,000 deaths *per* year in both Europe and the United States (*2*-*5*). The forensic determination of BAC is carried out by means of gas-chromatographic separation after headspace extraction (HS-GC) with either mass spectrometric (MSD) or flame ionization (FID) detection, using whole blood as the matrix of choice.

Operationally, beside AAI, cases a hospital and its clinical laboratory can be involved at various levels in the analysis of DUI, being in charge of sample collection alone or of the full confirmatory analysis. However, while the forensic analytical phase may be outsourced due to the need for specialized instrumentation and expertise, the resources of the preanalytical phase - from the personnel responsible for sample collection to the refrigerators used for sample storage - are often shared between diagnostic and forensic BAC testing. This has led to extensive discussions among laboratory specialists, which can be summarized as the need to identify, on one hand, the forensic defensibility characteristics of data produced by the diagnostic process and, on the other hand, the sustainability in a clinical environment of a specific process that guarantees the legal validity of the results (*6*-*11*).

To gain control over the preanalytical phase through operational procedures, it is necessary to understand the role and significance of various factors in determining BAC. Considering that the preanalytical phase in BAC accounts for 40% of the analytical result, compared to 20% for biological variability, one must have a comprehensive view of which factors are actually controllable (*12*). Notable contributions in this regard are provided by the scientific literature on forensic subjects (*13*). However, specifically for the clinical laboratory scientist, there is only one official document issued by the Clinical and laboratory standards institute (CLSI) (*14*). However, this document suffers from a series of limitations: it has never been revised since its initial publication in 1997, it is not currently officially available, and, most importantly, it only covers the preanalytical phase in three paragraphs (2.3 - 2.5) based on just six references.

The purpose of this literature review is therefore to provide the laboratory professional with the most comprehensive understanding of the factors determining the preanalytical phase of BAC, especially considering the coexistence of diagnostic and forensic processes in the clinical setting. A series of appendices to the text provides further insights into remarkable topics related with the BAC and the investigation thereof.

## Literature search

**Figure 1:**
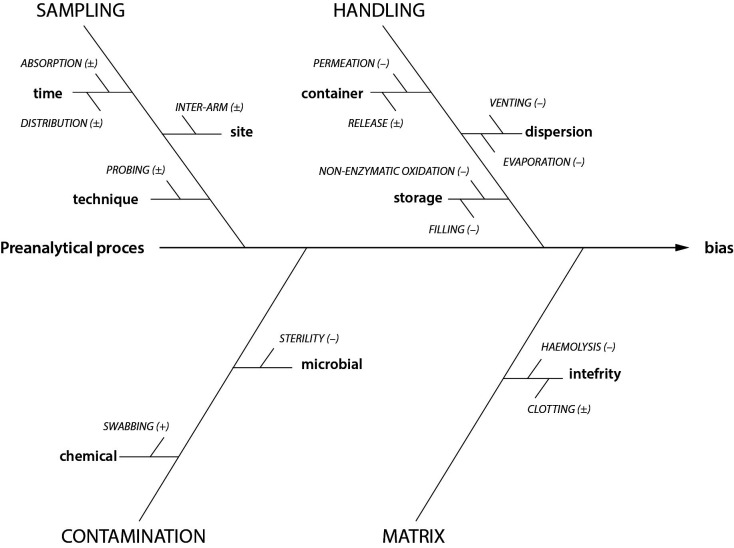
Fish-bone diagram of the major preanalytical factors of blood alcohol concentration (BAC) testing. To each factor (lower-case bold) is associated a source of bias (upper-case italics) with the sign indicated in parenthesis (the symbol “±” means that the exact sign depends on conditions as explained in the text).

Pubmed, Google Scholar and MEDLINE were searched for papers published until December 2022 with no restriction on language (see Appendix A). The search strategy based on a categorization of the subject according to a suitable model of the pre-analytics of a drug testing based on four major topics (namely, sampling, handling, contamination and matrix) is also represented in Figure 1. A typical query used for a preliminary search was as follows: (“blood alcohol” OR “blood ethanol”) AND “sampl*” AND (“stabil*” OR “stor*” OR “temperature” OR “contaminat*” OR “factor*”). The search was then refined within each topic adjusting by the use of more selective terms (*e.g.*, “stabiliz*”, “preserv*”, “additive”, “sealing”, “leak*” for the handling factor or “haemol*”, “clot*”, “lipem*”, “icter* for the matrix factor”). The literature search was further extended reviewing bibliography within each article issued before 1980s in order to retrieve any cited source that was eventually not electronically indexed (see Appendix A). Studies concerning *post-mortem* specimens or animals were excluded.

## Pre-analytical factors

### Sampling time

Ethanol is a very low molecular weight compound (46.07 g/mol and density of 0.789 g/mL at 20 °C) that can freely diffuse through the cell membrane (*13*). When ingested as a diluted water solution, it is absorbed *per* passive diffusion, first in the stomach and then extensively in the intestine (*13*). The peak of blood absorption depends on the timing of gastric emptying but not on the concentration or the volume of the consumed beverage due to the very large absorptive surface of the proximal small bowel (*15*, *16*). Accordingly, the time to reach body fluids equilibrium is erratic and depends on both the stomach emptying and the variations of the blood flow of the gut (*17*).

In general, the fasting peak blood is observed within 45-60 minutes after finished drinking, however in some subjects it may already be present by as early as 15 minutes or delayed to up to 120 minutes from ingestion (*13*). In this phase, where EtOH is equilibrating between blood and tissues, there is a negative veno-arterous bias (-Δ_V/A_) up to - 0.20 g/L in the same tributary area (*e.g.*, cephalic vein *vs.* radial artery), and a positive veno-venous bias (+Δ_V/V_) between proximal and distal tributary areas (*e.g.*, up to 0.06 g/L forearm *vs.* opposite foot) (*17*-*20*). Venous BAC is also affected by large erratic fluctuations caused by the equilibration phenomena (*21*).

In the post-absorption phase that begins about 90 minutes post-drinking, the veno-arterous bias changes the sign (+Δ_A/V_) and no veno-venous bias (Δ_V/V_≈0) is usually observed (*17*-*20*). In this phase, the venous BAC reflects the distribution equilibrium between blood and tissues, as in the brain where it exerts its psychoactive action. In this phase, if blood is recollected within 10 minutes from the previous sample, the resampling bias lays within the physiological random fluctuations (averaging ± 0.008 g/L and ± 0.010 g/L for venous and arterial blood, respectively) (*13*, *22*).

It should be noted that due to the kinetics of distribution to and from the tissues, the capillary blood shows an additive negative bias ranging by - 0.04 to - 0.06 g/L (*17*, *23*).

### Sampling site

The superficial veins laying in the antecubital fossa of the forearm represent the elective sites of routine phlebotomy, however they are characterized by remarkable topographical variability (both within- and between-subject) and more anastomoses than the deeper arteries (*24*). Thus, even when Δ_V/V_≈0, it can be observed with fairly large prevalence (almost 50%) an inter-arm bias (Δ_L/R_) that ranges between 0.008 g/L and 0.024 g/L with significant between-subject variability (coefficient of variation, CV≈60%) (*12*, *17*, *25*). Despite the size of this bias is almost the same before and after the absorption peak, its fluctuations are much larger in the absorption phase (*17*). Remarkably, this bias has unpredictable direction as it is unrelated with handedness, gender, pattern of the superficial veins, level of the blood gasses and haematocrit (*12*, *17*, *25*).

### Sampling technique

Since water content of blood and tissues shows very small within-subject variability (CV < 3.0%), any mechanical factor disturbing the perfusion flow can affect the equilibration of EtOH and in turn the BAC (*22*, *26*, *27*). For instance, a difficult positioning of an indwelling sampling device (catheter, butterfly needle) can increase up to five-fold the time-independent random BAC differences observed in the post-absorption phase (*22*).

The vasoconstriction of the tributary area induced by cooling (*i.e*., at 13-15 °C) doubles both the size and the duration of the Δ_A/V_ when the EtOH is administered by infusion, whereas the vasodilation (*i.e*., warming at 60 °C) halves only the size of the Δ_A/V_ (*28*). However, when EtOH is ingested, the duration (1 to 5 minutes) and the pressure applied to induce the venous stasis (60 to 100 mmHg) as well as the local ice-cooling of the skin do not have an effect over the BAC, while the body positioning (orthostatic reaction) and the physical activity produce a negative bias (*29*). This apparent contradiction between infused and ingested EtOH may eventually depend on the fact that the post-absorption route of distribution plays an unaddressed experimental and physiological role.

### Chemical contamination

Ethanol volatizes quickly from surfaces (the well-known “cooling effect” experienced after skin cleansing) with half-life on skin of 11.7 second, so that theoretically is necessary to expose 1000 cm^2^ of skin to 70% v/v alcoholic solution to achieve a BAC of 0.06 g/L (*30*).

Experimentally, a chemical contamination during sampling requires that the needle is suctioning while it is in direct contact with the alcoholic antiseptic (*e.g.*, pressing the soaked swab to stop bleeding while withdrawing the needle) (*31*-*33*). Otherwise, (*e.g.*, without pressing the soaked swab on the site of phlebotomy), with pre-evacuated tubes there is no contamination when the excess antiseptic is used (*i.e*., 2 mL) and alcohol is not allowed to dry off (*i.e*., 5 seconds waiting) (*34*). Likewise, the use of a syringe and thus of a controlled suction makes actually difficult to contaminate deliberately the specimens even pouring the alcohol directly onto the skin and inserting the needle shortly afterwards (*35*). Hence, if the correct amount of antiseptic is used (*i.e*., 1 mL) and it is allowed to dry off (*i.e.*, 1 minute), contamination has only 5% probability or less to happen (*36*, *37*). However, the spurious BAC due to a chemical contamination is erratic and unpredictable (*e.g.*, depending on the degree of swab squeezing), and values ranging from 0.005 g/L to up to 6.0 g/L have been reported (*33*, *35*, *36*).

It must be noted that when the experimentation involves inebriated subjects, the EtOH swabbing seems to give an average increase of 0.05 g/L of the BAC regardless of whether a pre-evacuated tube or a syringe was used (*38*-*40*). As already discussed for the mode of blood sampling, the discrepancy that occurs when the study involves inebriated subjects may rather be the effect of some uncontrolled experimental factor.

The effect of the chemical contamination depends on the analytical method used for measuring the BAC when skin disinfection is made with alcohol like isopropyl or amyl, since the spectrophotometric enzymatic assay based on yeast alcohol dehydrogenase (ADH) is highly selective for EtOH (*37*, *41*-*43*). Indeed, contaminating with isopropanol (*i.e*., 2-propanol) gives a negative bias when the BAC quantitation *via* the HS-GC uses this alcohol in place of the isomer n-propanol (1-propanol) as the internal standard (*44*).

### Microbial contamination

Even though the pre-evacuated tube used for blood sampling is a closed and sterile system that avoids environmental contamination during and after the blood withdrawal, the incorrect skin disinfection can be the source of microbes’ contamination during phlebotomy. Indeed, *Proteobacteria*, *Bacterotides* and *Staphylococcaceae* colonize the moisty skin of the antecubital fossa (*45*, *46*). Among the fungi, *Malassezia* predominates on *Candida* (*47*). All such microbes are able to synthesize anaerobically the EtOH *via* the fermentation (with production of small amounts of ethyl acetate as by-product), except for *Malassezia* that can only hydrolyse the fatty acids ethyl esters (*48*, *49*).

In a freshly collected blood sample, the contaminating microbes are in the disadvantage respect to the far more numerous erythrocytes for the uptake of glucose. Therefore, in such unfavourable conditions of substrates availability, the microbial ADH operates for the salvage pathway by reducing EtOH to restore nicotinamide adenine dinucleotide NAD^+^ from NADH + H^+^ (*50*-*52*). As the consequence, the bias of the BAC from a contaminated specimen is expected to be negative, except for the case of supplementation of the sample with extra-glucose as in a banked blood bag (*53*, *54*). Accordingly, the BAC of an EtOH-free (or below the detection limit) specimen remains unchanged regardless such factor as the use of sodium fluoride (NaF) preservative, the duration and temperature of the storage and the conditions of handling (*e.g.*, heat exposure, repeated uncapping and sampling) (*55*-*60*). Likewise, the environmental contamination of the blood matrix during the sampling and processing operations (*e.g.*, uncapping, decanting, pipetting) is unlikely to occur in a typical laboratory setting even when the sample is forcedly exposed or handled carelessly (*57*).

The NaF represents the major anti-microbial agent used for stabilizing the BAC and it has been recommended since very early in the scientific literature (*61*). At a concentration ranging within 200 mM and 300 mM (≈1% w/v), it causes the cell death within 24 hours, while at lower concentrations (*e.g.*, 100 mM) it can take up to 72 hours (*62*). Hence, at least within 24 hours the preservation of BAC is NaF-independent (*51*). Nonetheless, such microbes as *Pseudomonas* and *Serratia* can survive to NaF exposure up to 600 mM (≈2% w/v) (*52*). Hence, NaF acts as a second-line defense against contamination but is not superior to the primary sterility of the collection tube. Remarkably, in an ordinary tube used for glucose testing, the concentration of NaF is 60 mM (*i.e.*, 0.25% w/v or 2.5 mg/mL whole blood) and thus it is inadequate for preventing the growth of the microbes.

## Sample container

Ethanol is an organic compound whose octanol/water partition coefficient (logP_o/w_) lays close to - 0.3, therefore it cannot be adsorbed into the gel separator of clinical chemistry tubes as it requires logP_o/w_ > 3 (*63*-*66*). Accordingly, no adsorption bias is observed when the serum BAC is measured in plain or gel separator tubes (*67*, *68*). On the contrary, as a volatile organic solvent, the EtOH permeates the walls of tube made of single-layered polypropylene, but not the inner-layered polyethylene terephthalate and the glass borosilicate (*64*, *69*-*71*). In general, the permeation bias between a double-walled plastic and a glass tube rests within 1% of BAC regardless of the storage temperature (*69*, *72*). This condition is also unaffected by the extension of the contact surface of blood on the tube walls (*i.e.*, whether the tube is stored standing up or laying down) (*70*).

Occasionally, the gel separator can be the source of some chemical contamination, especially in the past due to the solvents used for the manufacturing of the rubber stoppers (unidentified substance) and gel separator of serum tubes (toluene, 1-butanol, ethylbenzene and xylene) (*73*-*75*). This was also found in whole blood sodium fluoride/oxalate (NaF/Ox) tubes containing traces of isobutylene (*76*). However, none of the above compound interferes with the spectrophotometric enzymatic assay of BAC. Conversely, chemical contaminants released from gel separator tubes and able to interfere with the HS-GC analysis carried out without the mass selective detector (MSD) were also reported recently. Particularly, these were a 1-propanol-like substance and ethyl chloride (chloroethane, C_2_H_5_Cl), both capable to positively bias the BAC as almost co-eluting with EtOH (*77*-*79*).

### Dispersion

The complete solubility of EtOH into water is due to the presence of the hydroxyl moiety on the very short carbon chain, so that the water/air partition coefficient (logP_w/a_) measured in distilled water does not significantly differ from whole blood and plasma (logP_w/a_≈3.33, logP_w/a_≈3.24 and logP_w/a_≈3.31, respectively) (*80*, *81*). Consequently, the evaporation bias of an uncapped serum or heparinized plasma tube resting at 22.1 - 25.1 °C temperature and 55% maximum air humidity averages - 3.0% within 30 minutes, - 5.0% after 1 hour and reaches a maximum of - 10.0% after 3 hours (*82*).

In a capped tube, EtOH collects into the air volume above the specimen (so-called “headspace”), reaching a concentration (*i.e.*, partial pressure) that only depends on the temperature as explained by the Henry-Dalton’s law. Accordingly, assuming that the distribution volume of EtOH contained in a 0.5 mL sample is about 2500 mL airspace at room temperature (20 °C), and that the headspace in a gas-tight sealed tube is about 1 mL when correctly filled, then the bias produced by venting once the headspace is less than -3% even at 40 °C (*80*, *81*, *83*, *84*). Therefore, a leak from the sealed cap occurring during the storage requires an extremely long period of time (*e.g*., 1 year) to reach a bias of - 1% (*85*).

As the loss of EtOH due to the partition in the headspace is negligible, there is no bias between 6 mL and 10 mL volume NaF/Ox pre-evacuated sterile tubes (*i.e*., different sample-to-headspace ratio) when these are correctly filled with the whole blood (*86*). However, when the NaF/Ox tube is partially filled so that the NaF reaches a final concentration of 2% or 5% w/v, the bias produced at room temperature by the headspace vent is - 3.0% and - 9.0%, respectively (*87*). This bias is due to the “salting-out” effect of the concentrated NaF in the matrix that increases the EtOH evaporation into the headspace (*81*).

Remarkably, for those longer chain alcohols (*e.g.*, propyl and butyl) used as the analytical internal standard in HS-GC, the salting-out is stronger because of their naturally lower water solubility (*88*, *89*). As a consequence, in case of partial filling the EtOH/internal standard peak area ratio in the sample obtained from 1% w/v NaF/Ox tube is fictitiously reduced giving rise to bias up to - 3% if no salt-saturated sample preparation is adopted (*87*, *90*-*92*).

### Sample storage

In healthy subjects, it can be found no ADH in a significant concentration either within erythrocytes or free in serum (*93*). However, the rate of acetaldehyde formation in ethylene diamine-tetra-acetic acid-anticoagulated (EDTA) whole blood containing EtOH steeply increases regardless of the addition of inhibitors (*e.g*., citric acid, iodoacetic acid, fluoride, aminoatriazole, azide and pyrazole) of oxidases that may attack very short-chained aliphatic compounds (*e.g.*, glyceraldehyde-3-phosphate dehydrogenase, catalase, ADH) (*94*). Therefore, a non-enzymatic oxidation (NEO) of EtOH exists and its kinetic depends on storage temperature, matrix oxygenation and haemoglobin concentration (see Appendix B) (*94*-*97*).

Based on studies whose conditions are compatible with sampling blood from a patient using a pre-evacuated sterile tube (*i.e.*, blood suctioned within by the vacuum), a whole blood sample can be delivered at room temperature and stored refrigerated (*i.e.*, - 20 °C) for up to 14 days with no forensically and diagnostically significant bias (see Appendix C) (*55*, *57*, *58*, *69*, *72*, *98*). However, in case of harsh handling of the specimen before storage or analysis (*e.g.*, exposure to elevated room temperature, high thermic excursion or external transportation without refrigeration), the bias increases up to - 0.20 g/L yet within the third day from collection (*56*). The prolonged heating surely enhances the kinetics of the NEO, but it is also likely to cause a loosening of the gas-tight cap through which allows the venting of the tube. In fact, even a short (< 20 min) but extreme heating (burning) gives negligibly biased of the BAC if the collection tube remains intact (*99*).

The negative bias tends to appear by 15 days up to 3 months depending on how much the temperature, oxygenation and time are actually affecting the kinetics of NEO (see Appendix C) (*95*). For instance, after 1 month at - 20 °C the bias averages - 6% when the headspace is 20% (*i.e.*, 1/5 of the tube volume) (*100*). However, venting the tube at least once before or during storage almost doubles the bias regardless of both the temperature (by - 10 °C to + 25 °C) and the duration (days to years) of storage (*55*, *58*, *60*, *101*, *102*). In general, a mild storage condition (*i.e.*, + 4 °C) gives a bias that is on average within - 0.04 g/L for one-year and - 0.20 g/L for three years (*103*, *104*). As the size of the storage bias correlates well with the storage time, the correlation between storage bias and the original BAC depends on the length of the time interval over which it is computed (*e.g.*, r = 0.80 up to 6 months, r = 0.23 up to 13 months *vs*. and r < 0.17 more than 5 years) (*55*, *58*, *60*, *100*).

Despite the lack of haemoglobin, the stored plasma shows - 7.8% bias after 2 months and up to - 25.2% after 5 months at - 20 °C (*105*). This bias is reduced to - 2% after 6 months if the sample is deproteinized by acid precipitation (*106*). Maybe, the loss of EtOH from plasma depends on the effect of residual iron within the matrix (also freed by the acid precipitation), as well as on the integrity of the gas-tight seal at low temperature.

Finally, an effectively preserving activity of BAC is observed in carbon monoxide-saturated whole blood where no significant bias occurs due to the blockage of oxyhaemoglobin formation (*107*). Comparable result can be achieved by deoxygenating the whole blood with bubbling nitrogen (*94*). This mechanism can be relevant for preserving the BAC in the samples used for quality control and proficiency testing, and it suggests that the smoking condition might act as a preanalytical factor interacting with other factors in the bias associated with the conditions of storage (*55*).

### Matrix integrity

The spectrophotometric enzymatic measurement of BAC in serum/plasma is based on the UV absorption peak of NADH + H^+^ at 340 nm, that is produced by the ADH according to a 1:1 stoichiometry during the oxidation of EtOH (*108*). This absorption peak is close to that produced by free oxygenated haemoglobin. However, the modern enzymatic assays use a 20-fold dilution to measure the BAC, so that no interference (*i.e.*, positive bias) is expected even with gross haemolysis (0.8 g/dL free haemoglobin) (*109*). It is likely that the haemolysis bias up to - 10% is caused by the oxidation of EtOH occurring before the enzymatic analysis, for instance as the consequence of the release of catalase from the erythrocytes (*109*-*111*). As this also accelerates the depletion of the cellular antioxidants that antagonize the NEO (see Appendix B), storing a sample with haemolysis causes a bias up to - 0.03 g/L within one week (*112*, *113*).

Ethanol can bind to hydrophobic sites of the albumin *via* the methyl group (*114*). Although this interaction can displace drugs like diazepam, warfarin and corticosteroids, the affinity constant toward albumin is actually large (K_d_ = 53.1 ± 3.1 mM or ≈2.4 g/L) (*115*-*117*). Since the formation of clots causes a minimal loss of protein and there is no sizable bound fraction of EtOH, the serum/plasma ratio of BAC is almost unity with very low variability (CV 0.01% to 0.03%) (*72*, *118*). However, since both the size and the water content (by 40% to 80% w/v) of clots is highly variable, the formation of clots within a whole blood specimen alters the distribution of water and makes unpredictable the bias caused by the inhomogeneity of the BAC (*119*, *120*). Thus, a specimen of whole blood with clots should be homogenized before the determination of BAC but the negative bias arising from the grinding process averages - 0.01 g/L (*121*).

## Other relevant factors related to the control of BAC

### Biosynthesis

In liver and kidney of humans, the mitochondrial oxidative decarboxylation of pyruvate produces acetaldehyde (*122*). Under hypoxic conditions, acetaldehyde is metabolized to EtOH by the cytosolic ADH in a redox reaction that converts the cofactor nicotinamide adenine dinucleotide from reduced (NADH + H^+^) to oxidized (NAD^+^) form (*122*). The frequency distribution of the BAC produced by this endogenous synthesis in ostensibly healthy and sober individuals is strongly left-skewed, with the 95^th^ and 99.4^th^ percentiles (age-, gender- and race-unrelated) corresponding to 0.012 g/L and 0.020 g/L respectively (*123*, *124*). To date, no individual value above 0.040 g/L has been reported (*125*).

Just in case of a rare clinical condition known as “auto-brewery” or “gut fermentation” syndrome (no more than 20 cases correctly identified in literature until 2021), usually associated with severe fungal infections, gastric resection or Crohn disease, the BAC can be as high as 0.5 g/L and up to 2.0 g/L under carbohydrates loading (*126*, *127*).

### Biochemistry

Because of the negligible binding to proteins and membranes, the distribution of EtOH follows the water content of blood components and thus it is partitioned between the serum/plasma fraction and the cytosol of the erythrocytes (with minor contributions from platelets and leukocytes) (*128*). Since the average water content of erythrocytes is ≈75% w/v (weight/volume), ≈85% w/v of whole blood and ≈95% w/v of serum/plasma, then the serum/whole blood as well as plasma/whole blood concentration ratio reflects the average water content of the blood components weighted by their relative abundance and is equal to 1.10-1.14 (*26*, *72*, *118*, *128*, *129*). This ratio varies more between- than within-subjects, and the CV < 3% is less than the biological variability of the haematocrit (CV≈5.5%) (*26*, *130*). Accordingly, the BAC measured in serum/plasma is unaffected by the haematocrit unless there is an extreme shift as in the polycythaemia or severe anaemia as during haemorrhage (*73*, *118*, *128*, *129*, *131*).

As water content rules the BAC, sizable deviations from the ratio of 1.10-1.14 can arise for a change in proteins and lipids content of serum/plasma (*129*, *132*). In this regard, only extreme alimentary, stress-related or genetic hyperlipidaemias can eventually reduce water content of serum by no more than 5%. On the contrary, much larger effect (in either directions) is expected for hyper- or hypoproteinaemia *via* the strong regulation of the oncotic pressure, that in turn affects the distribution of EtOH to the tissues (especially the skeletal muscle).

## Discussion

For the clinical laboratory, the BAC represents a significant organizational and cultural challenge due to its dual clinical and forensic significance. Even when not directly involved in the analysis of DUI cases - often outsourced to external laboratories for the need for specific instrumentation and expertise - the clinical laboratory may be involved in managing cases that, due to the circumstances in which they originated, can assume (often unpredictably) medico-legal relevance. It is here that the need for defensibility arises, that is, the ability to justify the forensic validity of the data, not as an added value but as an integral part of the diagnostic process. To this concern, it must be not disregarded the remarkable divide of magnitude between the allowable total error (TEa) set for the diagnostic BAC (between 20% and 9% of Clinical laboratory improvement amendments (CLIA) and Guidelines of the German Federal Medical Council (Rili-BAEK), respectively) and the combined measurement uncertainty recommended for the forensic BAC (within 4%) (*133*-*138*).

The preanalytical factors of the BAC (Figure 1) can generally be distinguished into two types: those related to the behaviour of EtOH within the patient’s body, which instantaneously determine the BAC at the time of phlebotomy, and those related to the behaviour of EtOH within the blood specimen, which determine the resulting BAC after phlebotomy. Remarkably, the factors of the first type (*i.e.*, time and site of sampling) have a limitedly controllable nature *via* the operative procedures (as in the case of a polytraumatized patient involved in a car accident whose exact time of last consumption is unknown). Notwithstanding their consideration falls within the logic of defensibility of the data, that for instance can be achieved through the postanalytical phase by building up an uncertainty budget derived from the knowledge of the associated bias and imprecision. The same applies to EtOH biosynthesis and biochemistry, the postanalytical control of which is perhaps the most significant expression of the dual clinical and forensic significance of the BAC, where a possible reference interval of endogenous EtOH forms the basis for a more realistic discussion on how to deal analytically with a zero-tolerance policy for EtOH consumption.

The factors of the second group (namely from sampling technique to matrix integrity), on the other hand, are operationally controllable. Therefore, they are the subject of more intense debate when the issue of coexistence between clinical and forensic preanalytics of the BAC is raised, because they determine the degree to which the operations need to be complicated to ensure the adequacy of the preanalytical process. In this regard, it should be noted that the preanalytics of the BAC is perhaps the most studied among laboratory tests and spans nearly a century of scientific publications (see the Appendix A). On the one hand, this is a favourable aspect because it indicates extensive characterization of the subject matter. On the other hand, it must be recognized that precisely because of this extensive characterization, the produced evidence has stratified through the evolution of knowledge and means that have accompanied the recent history of clinical chemistry. In other words, retrospective analysis often reveals discrepancies or incongruences among the evidence, making it difficult to grasp the correct indications provided by direct investigation or observation. In particular, this refers to the age-old question of whether it is necessary to collect and preserve the blood sample for forensic BAC separately from diagnostic BAC, that is, whether the preanalytical processes must necessarily be distinct.

In the investigation of preanalytics, a crucial aspect is represented by the relationship established between the experimental design and the factors effectively controlled by the experimenter (*139*). When considering the vast amount of literature on the preanalytics of the BAC, it is possible to explain and understand this only by taking into account the impact produced by the introduction of new blood collection devices, sterile and pre-evacuated, which have been increasingly used since the 1970s. These devices are the key that translates the control of preanalytical factors, as characterized by Smalldon, Brown and colleagues in their seminal works (even though they did not use such devices), into practice (*95*, *96*). If their experimental role is not explicitly taken into consideration (which is easy because they become part of routine activity for sample collection in studies), their presence or absence in a preanalytical study acts as a confounder (see Appendix C). Supporting this consideration, it is worth noting that no preanalytics study of the BAC has ever compared the use of these new devices with the previous technique based on blood collection with a syringe and dispensing into various pre-added but open tubes.

Given the above, there is no evidence to support the need for intensifying and differentiating the use of devices and procedures for AAI or DUI cases to mitigate bias for the latter if pre-evacuated and sterile collection tubes are used according to the correct phlebotomy procedures (*i.e.*, complete filling and mixing, vacuum sealing, and no haemolysis) (see Table 1). Indeed, under such conditions, the bias arising from intra- and extra-mural transport conditions, processing (*e.g.*, at room temperature for < 3 hours), short-term storage (*e.g.*, at + 4 °C for < 3 months), and long-term storage (*e.g*., at - 20 °C for < 3 years) adopted for diagnostics is compatible with the requirements for forensic BAC. This overall agrees with the conclusions reached by other authors through different paths, that there is no requirement for a specific preservative for forensic BAC, such as NaF (which, moreover, introduces additional complications like salting-out or haemolysis) (*140*, *141*).

**Table 1:** The “seven pillars” of a diagnostically reliable and forensically defensible unified preanalytics of blood alcohol concentration

**The “seven pillars” of a diagnostically reliable and forensically defensible unified preanalytics of BAC**
1. use non-alcoholic antiseptics for skin cleansing 2. change the site of phlebotomy instead of probing/manipulating 3. choose either heparin or EDTA anticoagulant 4. use only pre-evacuated sterile collection tubes of the smallest capacity (*i.e.*, 2.5 mL) 5. avoid air suction, tube venting and haemolysis before storage 6. freeze only for monthly or yearly storage 7. beware of vented or opened tubes especially for reanalysis
BAC - blood alcohol concentration. EDTA - ethylene diamine-tetra-acetic acid.

The only necessary special care relates to the use of non-alcoholic disinfectants for forensic BAC, which also benefits the diagnostics of AAI. In this regard, it should be remarked that guidelines provided by official laboratory medicine bodies such as the European federation of clinical chemistry and laboratory medicine (EFLM) do not ban alcoholic antiseptics for BAC request, but advice to take adequate time for drying off the alcohol before the venipuncture (*142*). Therefore, the present recommendation should be regarded as a reinforcement of that guidance, in a way that makes the preparation for blood collection for BAC analysis ‘error-proof’ against the pressures that a challenging operational context (*e.g.*, the Emergency Room) may exert on the correct timing of the procedure.

Since these disinfectants offer the same safety and usage procedures as alcoholic ones and are widely available in the market without significant budget impact, their substitutionary use (if not already in place) can be safely adopted for all blood sampling procedures (*24*). Therefore, the only necessary differentiation remains the adoption of a chain of custody for sample traceability whenever appropriate for the explicit medico-legal end of the requested BAC.

Therefore, it can be concluded that the execution of correct diagnostic preanalytical procedures guarantees both the diagnostic safety and forensic defensibility of clinical data, and no evidence contrary to a common preanalytical process can be found in the literature. It must be remarked that any deviation from these conditions magnifies the bias guaranteed by the use of pre-evacuated sterile devices (*e.g.*, opening of the tube for matrix sampling), compromising the integrity of the BAC, both forensic and diagnostic, regardless of the procedures adopted before and after its reanalysis.

## Appendix A – a historical note on the studies on the preanalytical phase of blood alcohol concentration

In the body of studies that make up the scientific literature on blood alcohol concentration (BAC) preanalytics, it is difficult to make a clear separation between the contributions of biochemical-clinical, forensic and pharmacological research. However, there is no doubt that forensic medicine has given a fundamental impetus to this field, certainly long before preanalytics was recognized as a subject of study in its own right within laboratory medicine. The systematic study of the preparation and storage of the sample, in fact, represents the way (documented since 1940) to give a scientifically authoritative answer to the disputes about the BAC measurement results often raised in cases of drunk driving (*35*, *61*, *143*, *144*).

As evident, this review is distinguished by the citation of a large number of articles originally written in German, many of which represent a unique source of evidence on certain aspects of the preanalytics of the BAC (see for instance references (*119*, *120*)). The journals that have published many of these articles, such as “Blutalkohol” (ISSN: 0006-5250, https://www.bads.de/wissen/fachzeitschrift-blutalkohol/) and “Deutsche Zeitschrift für die gesamte gerichtliche Medizin” (in later “Zeitschrift für Rechtsmedizin” and currently “International Journal of Legal Medicine”), played a leading scientific role in studies on the determination of BAC during the second half of the 21st century (*145*). It is therefore not surprising that the British Medical Association’s “Proceedings of the International Conference on Alcohol and Road Traffic” included scientific contributions written in German without the need for English translation.

## Appendix B – the mechanism of the non-enzymatic oxidation (NEO) of ethanol

The biochemical details of the non-enzymatic oxidation (NEO) are still unclear albeit there are several clues to the putative mechanism. First, the energy barrier to the formation of acetaldehyde (*i.e.,* activation energy) is only relatively higher in *ante-mortem* (≈25 kcal/mol) than in *post-mortem* (≈12 Kcal/mol) samples (*95*, *102*). This suggests that no enzyme is involved, even because the activation energy decreases almost tenfold when the headspace changes from practically zero to about 2/3 of the tube volume (*102*). Second, the kinetics of reaction respect to the ethanol is first or pseudo-first order (*i.e.,* concentration dependent) in *post-mortem* whilst it is zero-order (*i.e.,* concentration independent) in *ante-mortem* blood samples (*55*, *95*, *96*, *102*). Thus, the oxidation rate is constrained by the counteracting anti-oxidant defenses that are operating in the viable erythrocytes. Third, those agents that directly reduce the haeme (azide and nitrite) or block its oxidation (cyanide) or deoxygenate the matrix (nitrogen bubbling), abolish almost completely the oxidation of ethanol in whole blood even at room temperature (*94*, *96*). Fourth, the haematin alone is able to oxidize the ethanol although to a lower rate than the intact haemoglobin (*94*). Fifth, the oxidation rate is dependent on temperature (*112*).

Collectively, the evidences suggest that the NEO follows a Fenton catalysis mediated by haeme and involving hydrogen peroxide (H_2_O_2_) (*134*, *146*). In this regard, the hypoxic storage may increase the generation of reactive oxygen species like H_2_O_2_ because of the enhanced auto-oxidation of haemoglobin (with the formation of methaemoglobin) as well as the depletion of the cellular detoxifying mechanisms (*147*, *148*). Thus, it is likely that ethanol may be oxidized to acetaldehyde either directly *via* H_2_O_2_ and haeme (either as Fe^3+^ or Fe^2+^) as it occurs in the cytochrome (but with no further oxidation of acetaldehyde to acetic acid), or *via* a highly-reactive ferryl (Fe^4+^) haeme intermediate (*94*, *96*). The end of the catalysis would then depend on the consumption of the oxygen within the matrix rather than of haemoglobin. Theoretically, this should correspond to the loss of up to 0.2 g/L of ethanol according to the partial pressure of the oxygen in blood, a value that fit in the bias usually observed in long-term storage (*96*). It is clear then why freezing just slows down but does not stop the catalysis, venting the tube ignites new oxidative reactions *via* the residue haeme and the haemolysis before the storage prompts the oxidation *via* the premature depletion of the intracellular antioxidants.

## Appendix C – the experimental design and the stability of blood alcohol concentration

Since the early studies on the preanalytics of BAC, the term “stability” has been used to indicate the change in ethanol concentration occurring between sample collection and analysis (*51*, *58*, *60*, *61*, *65*, *82*, *95*, *96*, *101*, *107*, *112*, *144*). However, the exact attribution of a budget of bias to this term strictly depends on the experimental design by means of which it is measured the response of BAC under different conditions (*139*). In fact, the term “stability” should be used to indicate the bias arising from the spontaneous decay of ethanol within its matrix, and thus the amount loss just because of NEO (*96*). Accordingly, a “stabilized” specimen should be one where NEO was chemically inhibited (*107*).

By the point of view of the experimental design investigating the stability of BAC, the measured bias is the omnibus response built up by factors like condition (*i.e.*, temperature) and duration (*i.e.,* time) of storage, and the interactions of all these terms (*95*). However, as NEO starts as soon as the whole blood is collected, the effect is easily confounded by the underlying sterility condition of the matrix, whose effect also interact with time and temperature. As a consequence, there arise different if not conflicting estimates of “stability” bias because of incomplete factorization of the design (*i.e.,* incomplete attribution of the major variance components), particularly because of sterility issue (*143*, *144*, *149*-*151*). Otherwise, the “stability” bias arises as a trending (*i.e.,* systematically increasing) loss of ethanol, whilst other factors eventually acting as confounders (*e.g.,* loss by leakage) show a rather random behaviour.
